# Genomic evaluation of residual feed intake in US Holstein cows: insights into lifetime feed efficiency

**DOI:** 10.3389/fgene.2024.1462306

**Published:** 2024-11-11

**Authors:** P. Khanal, J. Johnson, G. Gouveia, A.T.H. Utsunomiya, P. Ross, N. Deeb

**Affiliations:** STgenetics, Navasota, TX, United States

**Keywords:** residual feed intake, dry-matter intake, energy-corrected milk, body weight, lactating cow, feed efficiency, genomic evaluation

## Abstract

Residual feed intake (RFI) is an important trait of feed efficiency that has been increasingly considered in the breeding objectives for dairy cattle. The objectives of this study were to estimate the genetic parameters of RFI and its component traits, namely, dry-matter intake (DMI), body weight (BW), and energy-corrected milk (ECM), in lactating Holstein cows; we thus developed a system for genomic evaluation of RFI in lactating Holstein cows and explored the associations of the RFI of heifers and cows. The RFI values were calculated from 2,538 first (n = 2,118) and second (n = 420) lactation Holsteins cows between 2020 and 2024 as part of the STgenetics EcoFeed^®^ program. Of the animals, 1,516 were heifers from the same research station with previously established RFI values . After quality control, 61,283 single-nucleotide polymorphisms were used for the analyses. Univariate analyses were performed to estimate the heritabilities of RFI and its components in lactating cows; bivariate analyses were then performed to estimate the genetic correlations between the RFI of heifers and lactating cows using the genomic best unbiased linear prediction method. Animals with phenotypes and genotypes were used as the training population, and animals with only genotypes were considered the prediction population. The reliability of breeding values was obtained by approximation based on partitioning a function of the accuracy of the training population’s genomic estimated breeding values (GEBVs) and magnitudes of genomic relationships between the individuals in the training and prediction populations. The heritability estimates (mean ± SE) of the RFI, DMI, ECM, and BW were 0.43 ± 0.07, 0.44 ± 0.04, 0.40 ± 0.05, and 0.46 ± 0.04, respectively. The average reliability of the GEBVs for RFI from the training and prediction populations were 44% and 30%, respectively. The genetic correlations for the RFI were 0.42 ± 0.08 between heifers and first lactation cows and 0.34 ± 0.06 between heifers and first and second lactation cows. Our results show that the genetic components of RFI are not fully carried over from heifers to cows and that there is re-ranking of the individuals at different life stages. Selection of animals for feed efficiency on a lifetime basis thus requires accounting for the efficiencies during animal growth and milk production as a lactating cow.

## 1 Introduction

Feed efficiency (FE) has a large impact on the profitability of the dairy industry as feed accounts for approximately 50% of the total farm cost (USDA, 2022). Improved FE could reduce the land use and other resources used for feed production (Lovendahl et al., 2018), which would help producers improve the overall production efficiency. In addition, improving FE improves the environmental sustainability of farming as more nutrients are directed toward milk production instead of being lost in manure and methane production (Basarab et al., 2013).

Residual feed intake (RFI) has been identified as one of the suitable traits for use in selection programs to improve FE (Stephansen et al., 2023). RFI is a heritable (Tempelman and Lu, 2020) trait and is defined as the difference between an animal’s actual feed intake and its expected intake calculated from different energy sinks (e.g., milk production, body weight, and changes in body weight) (Tempelman et al., 2015; VandeHaar et al., 2016). Efficient animals are those that consume less than expected and have negative RFI values, whereas inefficient animals consume more than expected and have positive RFI values. The reduced intake of low-RFI dairy cows has important implications for on-farm profitability; given the strong relationship between intake and methane emission (Mills et al., 2003), selection of low-RFI cows has also been described as an opportunity to positively impact methane emission reduction (Knapp et al., 2014).

Improvements in the net FE have been limited by the high costs and difficulties associated with measuring individual feed intake. Recording of individual feed intake is generally restricted to research farms and nucleus breeding herds, for which the available data are limited. Genomic selection allows the inclusion of important economic traits that are difficult to measure in breeding programs that could be cumulative across generations (Pryce et al., 2015; Li et al., 2020). RFI has been included in the lifetime net merit index used to evaluate dairy cows in the United States for total genetic merit in the national genetic evaluation (Li et al., 2020; VanRaden et al., 2021). Although genomic selection is an appealing method to estimate the breeding values of FE, collecting accurate phenotypes to create a large reference population and provide high-accuracy predictions on such a population is challenging (Pryce et al., 2015).

The cost of raising dairy heifers constitutes approximately 25% of the total production expenses on a farm, with the feed costs accounting for roughly 50% of the overall expenditure for heifer rearing (Akins and Science, 2016). Numerous studies have documented that selection for RFI in heifers can persist as they mature into cows (Davis et al., 2014; Macdonald et al., 2014). However, a study by Connor et al. (2019) revealed a modest phenotypic correlation of only 0.37 between the RFI of growing heifers and their subsequent RFI as lactating cows in the United States during the first 100 days of lactation. Furthermore, Pryce et al. (2014a) reported lower accuracy of genomic estimated breeding values (GEBVs) for the dry-matter intake (DMI) of cows when heifers were used as the reference population and higher accuracy when using lactating cows as the reference population.

To develop a comprehensive selection strategy for enhancing the overall FE in dairy farming, it is imperative to obtain accurate genetic and phenotypic correlation estimates between the RFI of growing heifers and lactating cows. Thus, the long-term goal of this study is to improve dairy FE. The main objectives of this study are as follows: i) to estimate the genetic parameters of RFI and its component traits in Holstein cows in the United States, ii) to develop a system for genomic evaluation for RFI in Holstein cows in the United States, and iii) to explore the associations between the RFI values of heifers and cows.

## 2 Materials and Methods

This study did not require approval from an Animal Care Committee because all required information was obtained from preexisting databases.

### 2.1 Data and phenotypes for heifers

The animal management, diets, data collection, and data quality control procedures were as described in Khanal et al. (2023). In brief, data were collected from 7,623 growing Holstein heifers across 204 trials conducted between 2014 and 2024 at the STgenetics Ohio Heifer Center (South Charleston, Ohio). Upon reaching 6 to 8 months of age, the heifers were moved to the feed conversion testing facility and placed in warm-up pens, where they began adaptation to a corn-silage-based test ration (Net energy gain; NE_G_ = 0.95 Mcal/kg Dry matter (DM); Crude protein (CP) = 12.0% DM). During adaptation, the heifers were evaluated for health and body size to establish groups of 40–64 animals exhibiting good health and adequate body sizes to freely consume feed from specialized feed bunks in the testing pens. Once a group was established, they were moved to a testing pen equipped with eight electronic feed bunks (GrowSafe Systems Ltd., Airdrie, AB, Canada). Once the heifers consumed the test rations for a minimum of 21 d, the feed intake and performance were measured daily for a minimum of 70 d. Throughout the adaptation and testing periods, the heifers were allowed *ad libitum* access to feed and clean drinking water. During each trial, the body weight (BW) was recorded using a chute weighing system (Tru-Test Inc., Mineral Wells, TX, United States) either biweekly (every 2 weeks) or twice during the first and last weeks of the testing period. For each animal, the DMI was recorded daily during the testing period. Individual feed intake was then computed using a subroutine of GrowSafe 4000E software, as described by Parsons et al. (2020). A linear regression of the serial BW on the day of the trial was calculated for each animal using JMP (SAS Inst. Inc., Cary, NC, United States) to determine the average daily gain (ADG) (Williams et al., 2011). The average DMI and metabolic BW (MBW) were determined as the averages of all daily DMI and MBWs over the measurement period, respectively. RFI was then computed for each trial as the difference between the actual and predicted DMI values using the following model:

$$y_{{DMI}_{h}}=µ+b_{1}\text{ ADG}+b_{2}\text{ MBW}+b_{3}\text{ age}+e_{{DMI}_{h}},$$

where $y_{{DMI}_{h}}$ is the average DMI over the trial period; µ is the overall mean effect for each trial; $b_{1}$ , $b_{2}$ , and $b_{3}$ are partial regression coefficients; ADG and MBW are the corresponding values averaged over the trial period; age is the average age of the heifer fitted as a covariate; and $e_{{DMI}_{h}}$ is the residual and considered as RFI_heifer_.

### 2.2 Data and phenotypes for lactating cows

Data were collected from 2,538 first (n = 2,118) and second (n = 420) lactation Holsteins cows between 2020 and 2024 at the STgenetics Ohio Heifer Center. Upon calving, the cows were placed in pens equipped with automatic milking systems (AMS; Lely Astronaut A5). Upon reaching 100 days of lactation, the cows were moved to two pens (225 head capacity per pen) equipped with 84 electronic feed intake bunks (GrowSafe Systems Ltd., Airdrie, AB, Canada). Individual animal records for the DMI, milk yield (MY), milk fat percentage (F%), milk protein percentage (P%), and BW were recorded daily from 100 to 240 days of lactation. Throughout the trials, the cows were fed a corn-silage-based ration (NE_l_ = 1.65 Mcal/kg; CP = 13.7%) and clean drinking water *ad libitum*. Only those cows that were determined to be ≥90% Holstein through genomic testing were included in this study, and all the cows were housed in free stall barns with no access to pasture.

The individual animal feed intake was computed using a subroutine of the GrowSafe 8000E software (process feed intakes), as described by Parsons et al. (2020). The MY, milk components, and BW data were recorded for each milking and transformed into daily values per cow using the AMS software. Energy-corrected milk (ECM) was then calculated as outlined by Orth (1992):

ECM=0.327* Milk yield+12.95* Fat Yield+7.2* Protein Yield.

If more than one lactation record was available for a given animal, only the records from the first lactation were used. The animal records were removed if an animal had less than 30 daily records of BW, DMI, or ECM available between 100 and 240 days of lactation. Then, the daily BW, DMI, and ECM records were included or excluded for individual animals based on residuals from the linear regression of BW, DMI, or ECM on the days of lactation for each animal if the values were more than three standard deviations (SD) above or below the mean residuals with one exception, i.e., the residual was greater or lower than three SDs for both DMI and MY in the same direction. This exception was allowed to include the obvious biological outliers in the data. Less than 5% of the daily BW, DMI, and ECM records were removed as outliers in this study, and these exclusions were random throughout the testing period. When these data were removed, the remaining values were used in the linear regression estimation of trait based on days of lactation using JMP. The average BW, DMI, and ECM values were determined as the mean of all daily BW, DMI, and ECM records, respectively, over the measurement period. Change in the body weight (∆BW) of a cow was calculated as the difference in BW between the start and end of the trial (Pryce et al., 2015). Across both primiparous and multiparous cases, there were a total of 2,538 unique cows. A detailed summary of the average BW, DMI, ECM, F%, and P% values is presented in Table 2. Of the cows analyzed, 1,516 animals also had previous RFI phenotypes as heifers.

The statistical model used to estimate the RFI of a lactating cow was as follows:

$$y_{{DMI}_{c}}=µ+\text{trial}+\text{lactation}+b_{1}\mathrm{DIM}+b_{2}\;{\mathrm{DIM}}^{2}+b_{3}age+b_{4}\;{age}^{2}+b_{5}BW+b_{6}ECM+b_{7}∆BW+e_{{DMI}_{c},}$$

where $y_{{DMI}_{c}}$ is the average DMI over the 140-d experimental period; µ is the overall mean; trial is the fixed effect of a trial (concatenation of pen, year, and season: 26 levels); lactation is the fixed effect of lactation (2 levels); DIM is a continuous covariate of the average days of lactation with regression coefficient $b_{1}$ ; ${\mathrm{DIM}}^{2}$ is the continuous covariate of the quadratic average days of lactation with regression coefficient $b_{2}$ ; age is a continuous covariate of the average age with regression coefficient $b_{3}$ ; ${age}^{2}$ is the continuous covariate of the quadratic average age with regression coefficient $b_{4}$ ; BW is a continuous covariate of the average BW with regression coefficient $b_{5}$ ; ECM is a continuous covariate of ECM with a regression coefficient of $b_{6}$ ; ∆BW is the continuous covariate of change in the body weight of a cow with regression coefficient $b_{7}$ ; and $e_{{DMI}_{c}}$ is the random residual effect that was considered RFI_cow_.

### 2.3 Genetic information

Animals from the research and commercial herds were genotyped with the STgenetics 70k customized chip (VM2; proprietary information). Quality control procedures were then applied by removing the sex chromosomes and single-nucleotide polymorphisms (SNPs) that had call rates of less than 95% or minor allele frequencies less than 5% and all animals with SNP call rates less than 95%. As a result, a total of 61,283 SNPs remained for the downstream analyses. Next, the genotypes were prephased using Eagle v2.4.1 (Loh et al., 2016). Then, minimac 4.1.0.2 (Das et al., 2016) was used to impute the missing genotypes. The imputed VCF files were then converted to plink files.

### 2.4 Estimation of variance components

Univariate analyses were performed to estimate the heritabilities of RFI and its component traits (DMI, BW, and ECM) for the lactating cows using LMT software (Boerner, 2022). The variance components were estimated using the following model:

$$y_{ijklm}=µ+{trial}_{i}+{lactation}_{j}+b_{1}{\mathrm{DIM}}_{k}+b_{2}{\mathrm{DIM}}_{k}^{2}+b_{3}{age}_{l}+b_{4}{age}_{l}^{2}+{animal}_{m}+e_{ijklm},$$

where $y_{ijklm}$ is the component trait of RFI (DMI, BW, and ECM) for each animal; µ, ${trial}_{i}$ , ${lactation}_{j}$ , $b_{1}$ , ${\mathrm{DIM}}_{k}$ , $b_{2}$ , ${\mathrm{DIM}}_{k}^{2}$ , $b_{3}$ , ${age}_{l}$ , $b_{4}$ , and ${age}_{l}^{2}$ are the same as the variables explained earlier; ${animal}_{m}$ is the random additive genetic effect that has been assumed to be normally distributed with animal ∼N (0, **A** $\sigma_{a}^{2}$ ), where **A** is the pedigree relationship matrix built on pedigree traced back 10 generations and $\sigma_{a}^{2}$ is the estimated additive genetic variance; and $e_{ijklm}$ is the vector of random residuals with e ∼ N (0, **I** $\sigma_{e}^{2}$ ), where $\sigma_{e}^{2}$ is the residual variance and **I** is the identity matrix. In the pedigree, the base population was considered to be unrelated.

The RFI was modeled only as a function of the genetic effects (a), second residual (e), and overall mean (µ) to estimate the variance components because the other effects are already accounted for in the estimation of the RFI.

### 2.5 Genomic prediction of lactating cows

Genomic prediction was performed in a two-step approach. The variance components obtained from the pedigree relationship matrix were fixed, and the genomic relationship matrix **G** was created according to VanRaden (2008) and used to estimate the genomic breeding values. Here, **G** = $\frac{\left(M-2\Pi \right){\left(M-2\Pi \right)}^{\prime }}{2\sum_{j=1}^{m}p_{j}\left(1-p_{j}\right)}$ , where Π is a matrix with all the elements in column *j* containing the frequency of the second allele for the SNP marker *j* in the base population. **M** represents the *n*_*g*_ × *n*_*m*_ matrix of *n*_*m*_ genotypes for the *n*_*g*_ genotyped animals, which are coded as 0, 1, and 2 for the number of copies of the reference or first allele for each genotype. The predictions of the GEBVs for the training population (lactating cows with genotype and phenotype) were obtained by solving the mixed model equations. Once the GEBVs of the training population were estimated, the GEBVs of the prediction population (lactating cows with only genotype but not phenotype) were estimated as explained previously by VanRaden (2008). In brief, ${\hat{u}}_{p}$ **=** $G_{\mathrm{PT}}G_{\mathrm{TT}}^{-1}{\hat{u}}_{T}$ , where ${\hat{u}}_{p}$ is the vector of breeding values of the prediction population, ${\hat{u}}_{T}$ is the vector of GEBVs of the training population, $G_{\text{PT}}$ is the relationship between the training and prediction populations, and $G_{\text{TT}}^{-1}$ is the inverse of the genomic relationship of the training population. The reliability was then calculated as follows:

$$\text{rel}\left({\hat{u}}_{p}\right)\;{=G}_{\text{PT}}G_{\text{TT}}^{-1}\left[G_{\text{TT}}\sigma_{a}^{2}-C_{22}\sigma_{e}^{2}\right]G_{\text{TT}}^{-1}G_{\text{TP}},$$

where $C_{22}$ is a part of the inverse of the left-hand-side matrix of the mixed model equation (Taylor, 2014).

To illustrate the validity of the predicted breeding values on other populations with different relatedness to the training population, we selected four groups of prediction populations from commercial partner herds, with 5,000 animals in each group based on genetic relationship with the training population. Here, Group 1 includes animals that share the same sires as those of the training population, Group 2 includes animals that only share the same grandsires but not sires as those of the training population, Group 3 includes animals that only share the same great-grandsires but not sires and grandsires as those of the training population, and Group 4 includes animals that do not share sires, grandsires, or great-grandsires as those of the training population. To investigate the impact of genetic distancing from the training population on the reliabilities of the predicted breeding values, we estimated the number of close relatives in the training population for each animal in the defined groups. We assumed close relatives with genomic relationship values of 0.45 or more, which is equivalent to a first-degree relationship (e.g., 0.5 for parent–offspring or full siblings), by accounting for an estimation error of ±0.05. The associations between reliability and estimated number of close relatives was then evaluated through a non-linear regression analysis.

### 2.6 Association between RFI of heifers and lactating cows

Bivariate analyses (RFI_heifer_, RFI_cow_) were performed among the animals having phenotypes of both RFI_heifer_ and RFI_cow_ to estimate the genetic correlations between i) RFI of heifers and cows with first lactation (n = 1,360) as well as ii) RFI of heifers and cows with both first and second lactations (n = 1,516). The variance components from the bivariate analysis were obtained using the following model:

$$y_{T}=X_{T}b_{T}+Z_{T}g_{T}+e_{T},$$

where $y_{T}$ is the matrix of vectors of RFI_heifer_ and RFI_cow_; $b_{T}$ is the matrix of fixed effect solutions (mean of each trait); $X_{T}$ is the incidence matrix for the fixed effects for both traits; $Z_{T}$ is the incidence matrix mapping random animal effects for RFI_heifer_ and RFI_cow_; $g_{T}$ consists of the random animal effects for RFI_heifer_ and RFI_cow_; and $e_{T}$ is the vector of random residuals for RFI_heifer_ and RFI_cow_. The additive effects were normally distributed as N (0, **A** ⊗ **K**), where **K** is a 2 × 2 matrix of additive genetic (co)variances between RFI_heifer_ and RFI_cow_, and **A** was constructed in the same way as in the univariate analysis. The residual variance is V ( $e_{T}$ **)** = **R** ⊗ **I**, where **R** is a 2 × 2 matrix of error (co)variances between RFI_heifer_ and RFI_cow_ and **I** is the identity matrix. The standard errors (SE) of the variance components were estimated as the SD of 100,000 multivariate normal random vectors drawn from a multivariate normal distribution with a mean vector based on the estimated variance components and variances from the asymptotic (co)variance matrix obtained from the inverse of the average information matrix (Meyer and Houle, 2013).

## 3 Results and discussion

### 3.1 Phenotypic data

**FIGURE 1:**
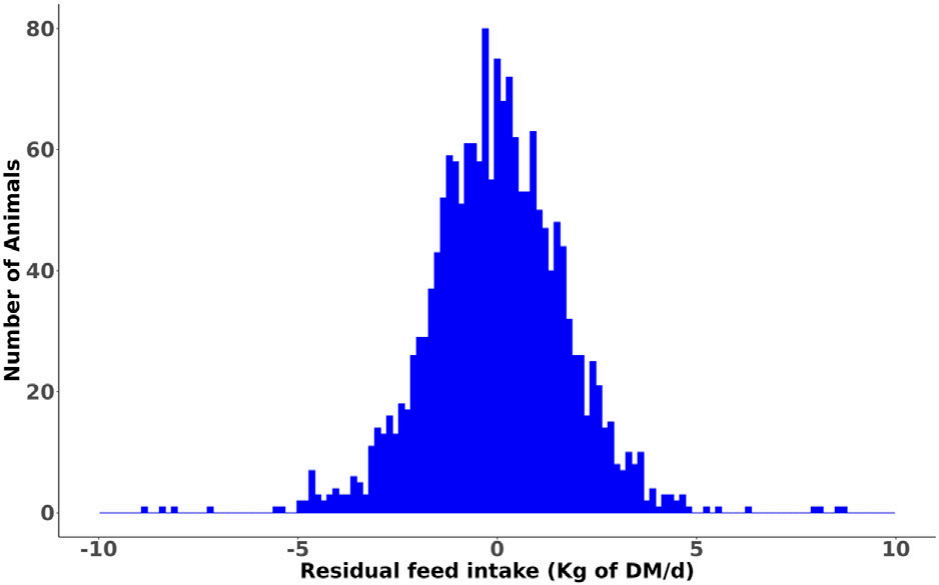
Distribution of the phenotypic residual feed intake (RFI) values of lactating cows.

Summary statistics of the RFI of heifers and its component traits averaged over the trial period are summarized in Table 1, and the corresponding data for the lactating cows grouped by parity are presented in Table 2. A histogram of the RFI for all cows (Figure 1) shows that RFI is normally distributed and ranges from −9.75 to 9.67 kg/d. The observed phenotypic SD of RFI_cow_ was 1.85 kg of DM/d (8.5% of average DMI), which is in agreement with the values reported by Connor et al. (2013) (1.63 kg of DM/d or approximately 7% of average DMI) and Cavani et al. (2022) (1.99 kg of DM/d or approximately 7% of average DMI) in US Holstein lactating cows. Notably, the SD of RFI in lactating cows is more than twice the phenotypic SD of RFI_heifer_ (0.86 kg of DM/d, Khanal et al. (2023)).

**TABLE 1:** Summary statistics of the residual feed intake (RFI) values of heifers and the component traits averaged over the trial period.

Trait^a^	Mean	Min	Max	SD
RFI (kg of DM/d)	0	−4.69	4.21	0.76
DMI (kg/d)	7.98	3.14	14.42	1.54
Age (d)	307	206	437	43
MBW (kg)	60.0	35.22	84.59	7.5
ADG (kg/d)	1.09	0.24	2.70	0.24

^a^ DMI, dry-matter intake; MBW, metabolic body weight; ADG, average daily gain.

**TABLE 2:** Summary statistics by parity of the component traits of residual feed intake of lactating cows averaged over the trial period.

	Traits^a^	Mean	Min	Max	SD
Parity 1					
	DMI (kg)	20.0	12.0	28.8	2.25
	BW (kg)	677	444	915	75
	ECM (kg)	35.1	4.6	53.0	6.7
	∆BW (kg)	0.47	−9.16	9.59	1.09
	Fat (%)	4.27	2.54	6.23	0.57
	Protein (%)	3.33	2.77	3.89	0.16
	Age (d)	945	690	1,377	87
Parity 2					
	DMI (kg)	23.9	16.1	36.6	2.81
	BW (kg)	749	559	934	67
	ECM (kg)	41.4	15.7	63.2	8.0
	∆BW (kg)	0.32	−3.84	9.81	0.81
	Fat (%)	4.33	2.77	6.48	0.67
	Protein (%)	3.31	2.83	3.83	0.16
	Age (d)	1,435	916	1918	152

^a^ DMI, dry-matter intake; BW, body weight; ECM, energy-corrected milk; ∆BW, change in body weight.

To illustrate the significant level of variance in the RFI within our cow population and highlight the potential value of selecting high-performing animals, we compared the top and bottom 10% of cows for RFI, as shown in Table 3. The average RFI for the most efficient and least efficient first-parity cows were −2.26 kg and 2.37 kg of DM/d, respectively (difference of 4.6 kg of DMI/d/cow), and those for second-parity cows were −2.43 kg and 2.61 kg of DM/d, respectively (difference of 5.0 kg of DMI/d/cow). These results show the large phenotypic variations in RFI that could be used for genetic selection among Holstein cows. As expected, there was a significant difference (*p* < 0.001) in DMI between the most and least efficient cows (17.30 vs. 23.30 kg/d for first lactation and 20.40 vs. 27.50 kg/d for second lactation); however, there were no differences in ECM, BW, and ΔBW values between the cows with divergent RFI. This result is attributed to the fact that these variables were already accounted for in the model to estimate the RFI. There were no differences in the MYs, fat and protein concentrations, and ages of first calving between cows with divergent RFI. Similar results have been reported for heifers analyzed at the same research station (Khanal et al., 2023). These results demonstrate the independence of RFI with respect to production, body size, and growth performance as well as the potential ability to select for improved FE independent of all other important production traits. However, it should be noted that the model used to derive RFI_cow_ in this study did not include all the potential energy sinks, such as physiological growth in pregnancy, body condition score, change in the body condition score, or energy spent through locomotion or movement.

**TABLE 3:** . Characteristics in terms of average (SD) of 10% of the lactating cows across lactations with the lowest (most efficient) and highest (less efficient) residual feed intake (RFI) rankings.

	Lactation 1		Lactation 2	
Trait^a^	Lowest 10% (n = 124)	Highest 10% (n = 131)	Lowest 10% (n = 45)	Highest 10% (n = 39)
RFI (kg of DM/d)	−2.26 (1.05)	2.37 (0.86)	−2.43 (1.15)	2.61 (1.10)
DMI (kg/d)	17.30 (2.11)	23.30 (1.89)	20.40 (1.97)	27.50 (2.54)
Milk yield (kg)	30.84 (6.31)	31.91 (6.41)	36.71 (6.00)	38.15 (7.58)
Fat yield (kg)	1.37 (0.31)	1.30 (0.29)	1.62 (0.26)	1.59 (0.37)
Protein yield (kg)	1.02 (0.20)	1.06 (0.21)	1.20 (0.19)	1.24 (0.25)
ECM (kg)	34.40 (7.84)	35.5 (7.07)	40.7 (7.34)	40.8 (8.16)
BW (kg)	684 (117)	679 (69)	713 (67)	774 (55)
∆BW (kg)	0.47 (1.98)	0.47 (0.68)	0.14 (0.53)	0.22 (0.82)
AOFC (days)	760.97 (78.20)	758.92 (78.15)	854.87 (167.55)	850.16 (114.81)

^a^ DMI, dry-matter intake; ECM, energy-corrected milk; BW, body weight, ∆BW, change in body weight; AOFC, age of first calving.

### 3.2 Genetic parameters

The variance components, heritability, and SE estimates from the univariate analyses of RFI_cow_, DMI, ECM, and BW are presented in Table 4. The estimated heritability of DMI was 0.44 ± 0.04, similar to those of other FE studies that range from 0.28 to 0.57 for different cattle breeds (Manzanilla Pech et al. (2014) for Dutch Holstein; Negussie et al. (2019) for Nordic Red; Khanal et al. (2022) for US Holstein). The estimated heritability of ECM was 0.40 ± 0.04, which was within the range of results reported in the literature for Holstein cows as ranging from 0.2 (Manzanilla Pech et al., 2014) to 0.46 (Khanal et al., 2022). Similarly, the estimated heritability of BW was 0.46 ± 0.04, which was in agreement with previous results reported by Manzanilla Pech et al. (2014) (0.38) and Khanal et al. (2022) (0.67). Lastly, the estimated heritability for RFI_cow_ was 0.43 ± 0.08, which aligns with the result of 0.36 ± 0.06 reported by Connor et al. (2013) across the entire lactation.

**TABLE 4:** Heritability estimates (±SE) of the residual feed intake (RFI) and its component traits in lactating Holstein cows.

Trait^a^	Additive genetic variance	Residual variance	Heritability estimate
RFI (kg of DM/d)	1.16 ± 0.20	1.55 ± 0.13	0.43 ± 0.07
DMI (kg)	2.65 ± 0.66	3.38 ± 0.44	0.44 ± 0.04
ECM (kg)	21.00 ± 5.92	30.94 ± 4.13	0.40 ± 0.05
BW (kg)	2403.72 ± 152.14	2836.34 ± 125.33	0.46 ± 0.04

^a^ DMI, dry-matter intake; ECM, energy-corrected milk; BW, body weight.

It should be noted that the RFI in the current study was based on average feed intake from all records collected during the experimental period, which decreased the noise across the testing period and likely yielded a slightly higher heritability. Indeed, weekly heritability estimates of the RFI in US Holstein cows reported in previous studies (Lu et al., 2015; Li et al., 2017, 2020) were lower and ranged from 0.14 to 0.29. Moreover, our data were collected from a single research station, as opposed to multiple research stations in other studies, which could be another reason for having less noise in the data and higher heritability. Heritabilities of the lactation-wise (or averaged) measures should be greater than those of their individual daily or weekly data, especially if the genetic and permanent environmental correlations between different DIMs for the corresponding traits deviate substantially from unity (Khanal et al., 2022). Different studies (Manzanilla Pech et al., 2014; Li et al., 2017; Negussie et al., 2019) have reported that the genetic correlations for DMI, ECM, and RFI across different DIMs deviate substantially from unity. The moderate heritability of RFI_cow_ in our study suggests that the RFI can be used in dairy cattle breeding to improve FE.

### 3.3 Genomic prediction of lactating cows

**FIGURE 2:**
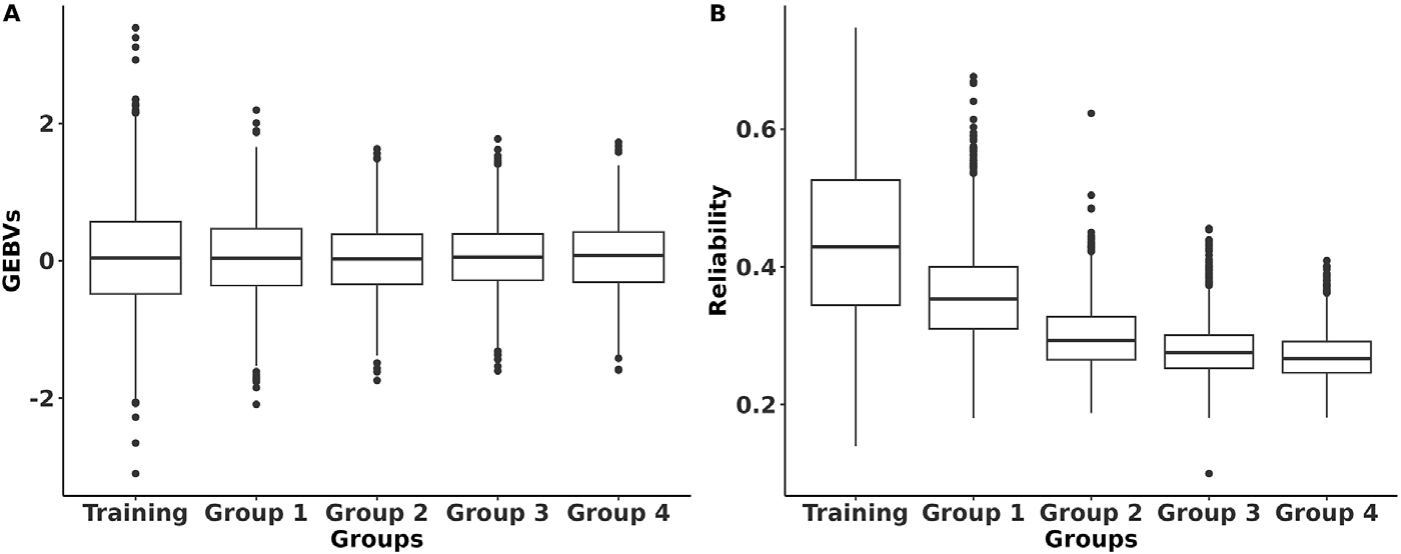
Distribution of the genomic estimated breeding values (GEBVs) and their reliabilities of RFI for Holstein lactating cows for different population groups. Training refers to the population with both genotypes and phenotypes; Panel **(A)** Group 1 includes animals that share the same sires as those of the training population Panel **(B)**; Group 2 includes animals that only share the same grandsires but not sires as those of the training population; Group 3 includes animals that only share the same great-grandsires but not sires and grandsires as those of the training population; Group 4 includes animals that do not share sires, grandsires, and great-grandsires as those of the training population.

**FIGURE 3:**
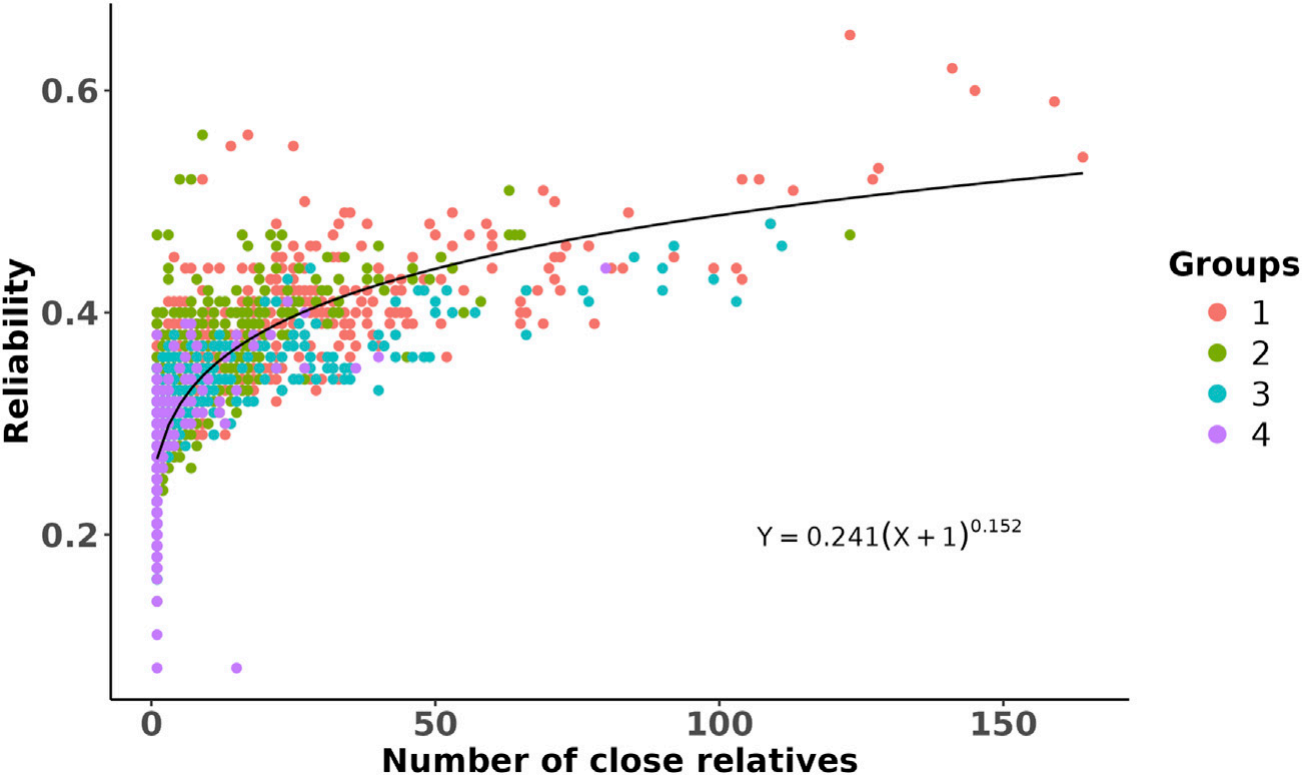
Scatter plot of the reliability of genomic prediction as a function of the number of close relatives that the predicted animals had with the training population. Each dot is an animal from a given prediction group (represented by different colors and as described in Figure 2), and its close relatives were defined on the basis of genomic relationship >0.45. The line shows a non-linear regression fitted to the data by assuming a power curve model.

The GEBVs and their reliabilities for RFI_cow_ were estimated for different groups of animals with different genetic distances between the training and predicted populations. The distributions of the GEBVs and reliabilities are presented in Figure 2. The SD and range of GEBVs of the training population were higher than those of different groups of the prediction populations (0.68 kg vs. 0.57 kg and −2.14 to 3.14 kg vs. −2.23 to 2.37 kg). The average reliability of the training population was 14% higher than that of the prediction populations. As expected, the average reliability of the prediction populations decreased from Group 1 that shared sires with the training population to Group 4 that was at least four generations removed from the genetic base of the training population. The decrease in reliability from Group 1 to Group 4 was due to the increase in relationship distance with the training population. Lower reliability for animals that were distant from the reference population was also reported by Li et al. (2020) in US Holstein bulls and Khanal et al. (2023) in US Holstein heifers. The reductions in average reliability compared to the training population were 18.2%, 31.8%, 36.4%, and 38.6% for Groups 1–4, respectively (Figure 2). These reductions were explained adequately by genetic distancing with the training population (Figure 3). Despite the small effective population size of the Holstein breed (Makanjuola et al., 2020; $N_{e}$ = 43 to 66 individuals) that results in high population-wide linkage disequilibrium and common haplotypes across many generations, the results illustrate the value of including a contemporary population in the training set for accurate predictions of the breeding values.

Owing to the limited data size, the average genomic reliability of RFI for the prediction population in our research was slightly lower (∼30%) than the current traits included in the lifetime net merit (NM$) index for Holstein cattle in the US (VanRaden et al., 2021). Despite the limited data size, the average genomic reliability of RFI in our study was comparable to those reported in Australia (∼40%) and US (∼18%) national genetic evaluations for Holstein cows (Pryce et al., 2014b; Li et al., 2020; Gaddis et al., 2021). The size of the training population and its relationship with the prediction population play significant roles in the reliability estimates. Therefore, efforts should focus on increasing the number of individuals in the training population, i.e., collecting more feed intake data. Collection of feed intake data should be a continual effort to prevent reductions in genomic prediction reliabilities owing to the distant relationships between currently recorded cows and future commercial animals.

### 3.4 Association between RFI of heifers and lactating cows

To evaluate the potential genetic differences in RFI values among heifers and different lactations of cows, we estimated the genetic correlations between the RFI of heifers and first lactation cows and the RFI of heifers and all cows (both first and second lactations). The estimated genetic correlations for RFI were 0.42 ± 0.08 between heifers and first lactation cows as well as 0.34 ± 0.06 between heifers and all cows. The genetic correlations in our study were lower than those reported by Pryce et al. (2015) and Bolormaa et al. (2022) in Australian cattle populations (0.67 and 0.47). However, both research groups reported high SEs, where the mean value indeed decreased with the addition of more animals in the analysis conducted by Bolormaa et al. (2022). Connor et al. (2019) reported a phenotypic correlation of 0.37 between the RFI values of growing heifers and cows in the US.

Our results show that the underlying genetic components of RFI are not fully carried over across the stages of life and that there is reranking of individuals when comparing the RFI values for heifers and lactating cows. Out of the 1,516 animals that had phenotypes of both RFI_heifer_ and RFI_cow_, we ranked heifers as the least (upper 10%; n = 155), medium (between 80%; n = 1,210), or most efficient (lower 10%; n = 151) according to the breeding values of RFI_heifer_. We found that 20.5%, 75%, and 4.5% of the most efficient heifers were ranked as most, medium, and least efficient as lactating cows, respectively. Conversely, 3%, 72%, and 25% of the least efficient heifers were ranked as most, medium, and least efficient as lactating cows, respectively. In essence, when examining the two extreme groups, only 4.5% of the high-performing group as heifers were classified within the bottom 10% as cows, while 3% of the least efficient animals as heifers were identified to be among the top 10% as cows. Similar results of reranking were reported in previous studies (Macdonald et al., 2014; Connor et al., 2019). Our results show that the genetic components of RFI of the heifers were not fully carried over as they matured into lactating cows. This indicates that evaluating the FE over a lifetime requires consideration of the varying energy needs at different life stages. Therefore, to select individuals based on the lifetime feed conversion efficiency, a comprehensive study involving the RFI of growing heifers and lactating cows is necessary.

### 3.5 Implications

Previous research (Tempelman et al., 2015; VandeHaar et al., 2016) has indicated RFI as a trait for selection as it has a direct impact on two major issues affecting the dairy industry today, namely feed cost and greenhouse gas emissions. The findings from our research support the conclusion that low-genomic-RFI dairy females were found to have reduced DMI with no impacts on other economically relevant traits. Given the strong associations between DMI and CH_4_ emissions (de Haas et al., 2012; Richardson et al., 2021), reduction of the DMI with no impact on productivity will result in reduced CH_4_ emissions from the animals. Utilizing equations published by the Intergovernmental Panel on Climate Change (IPCC 2019), improving the RFI of cows and heifers by 1 SD of GEBVs (RFI_heifer_ SD = 0.28 kg/d; RFI_cow_ SD = 0.57 kg/d) could reduce emissions by 422 kg of CO_2_ across their lifetime. Therefore, selection for RFI has significant implications for helping the industry achieve various emission reduction targets.

Reduced DMI achieved from selection for RFI also aids in driving on-farm profitability as the feed costs account for over 50% of the total input costs (USDA, 2022). By reducing the input costs and maintaining the output revenues, farmers can directly impact their net profits. If a farmer achieves a 1 SD improvement in heifer and cow RFI, they can reduce feed costs by $251.00 per animal based on the ration costs of US$263/ton DM for heifers and US$381/ton DM for cows, assuming a heifer rearing period of 580 d and productive life of 854 d for cows. While the lactating cow period has a higher economic impact on the net profit of a farm, ignoring the feed costs associated with heifer RFI would reduce the lifetime feed cost savings by US$ 38.70 per cow. Accordingly, by combining selection for both heifer and cow RFI, producers can maximize their feed and greenhouse gas savings while continuing to drive selection for output traits, such as MY as well as fat and protein percentages, for sustainable farming.

## 4 Conclusions

We explored the genetic basis of RFI and estimated its genetic parameters and component traits for Holstein cows in the US. The heritability of RFI of lactating cows was high, which indicated the possibility of selection of efficient animals. The mean reliability of RFI of lactating cows was low; however, we expect that this will improve as we strategically increase the number of RFI phenotypes from the relatives of elite bulls that have greater contribution to the training population. We found a low genetic correlation of RFI between cows and heifers; this indicates that the underlying genetic control of RFI is not fully carried over to different stages of life owing to differing energy requirements. We would recommend considering the RFI of growing heifers in addition to those of lactating cows in the genetic evaluation system to select highly feed-efficient animals on a lifetime basis.

## Data Availability

The datasets presented in this article are not readily available because the feed efficiency databases maintained by STgenetics are not publicly available. Requests to access the datasets should be directed to joseph.deeb@stgen.com.
